# Ubiquitination and ALL: Identifying FBXO8 as a prognostic biomarker and therapeutic target

**DOI:** 10.3389/fimmu.2025.1554231

**Published:** 2025-05-01

**Authors:** Wei Xian, Yinting Chen, Shuiqing Yu, Zhitao Ye, Yu Zhang, Danlin Yao

**Affiliations:** ^1^ Department of Pediatric Allergy, Immunology and Rheumatology, Guangzhou Women and Children’s Medical Center, Guangzhou Medical University, Guangzhou, Guangdong, China; ^2^ Department of Hematology, Shenzhen Hospital, Southern Medical University, Shenzhen, China; ^3^ School and Hospital of Stomatology, Guangdong Engineering Research Center of Oral Restoration and Reconstruction & Guangzhou Key Laboratory of Basic and Applied Research of Oral Regenerative Medicine, Guangzhou Medical University, Guangzhou, China; ^4^ Department of Nephrology, Guangzhou Women and Children’s Medical Center, Guangzhou Medical University, Guangzhou, Guangdong, China; ^5^ Department of Pediatric, Zhujiang Hospital, Southern Medical University, Guangdong, Guangzhou, China

**Keywords:** acute lymphoblastic leukemia, ubiquitination, FBXO8, prognostic model, immune microenvironment

## Abstract

**Background:**

Acute lymphoblastic leukemia (ALL) is a hematological malignancy with high survival rates in children; however, certain high-risk subtypes pose significant challenges due to poor prognosis and frequent relapse. Ubiquitination, a post-translational modification critical for protein regulation, has been implicated in various cancer processes, yet its role in ALL remains poorly understood.

**Methods:**

Using the TARGET database, we identified molecular subtypes of ALL through consensus clustering based on ubiquitination-related genes (URGs). A nine-gene prognostic model was constructed using LASSO and Cox regression analyses. The immunological landscape variations between high- and low-risk groups were assessed using immune cell infiltration analysis and functional enrichment studies. *FBXO8* was further explored through functional experiments *in vitro* and *in vivo*.

**Results:**

Four ALL subtypes with distinct survival outcomes were identified, with Cluster D representing the high-risk group. Patients were divided into high- and low-risk groups using the nine-gene predictive model, and *FBXO8* was found to be a significant protective factor. According to immune landscape analysis, high-risk groups had an immunosuppressive microenvironment that was correlated with *FBXO8* expression and marked by an increase in regulatory T cells and M2 macrophage infiltration. *In vitro* functional assays, *FBXO8* knockdown notably enhanced cell proliferation and suppressed apoptosis in ALL cells. In *FBXO8*-knockdown mouse models, *in vivo* investigations demonstrated increased tumor growth, reduced apoptosis, and diminished survival rates.

**Conclusion:**

This work identifies *FBXO8* as a crucial therapeutic target and prognostic biomarker for ALL. Knockdown of *FBXO8* led to the suppression of apoptosis and increased tumor growth, suggesting potential therapeutic applications. These results highlight the need for more investigation into ubiquitination-related pathways and offer important new insights into high-risk ALL.

## 1 Introduction

Acute lymphoblastic leukemia (ALL) is a malignant tumor arising from lymphocyte progenitor cells, characterized by rapid progression and a high fatality rate (1, 2). This blood and bone marrow cancer frequently causes extensive organ damage and, without timely treatment, can lead to death within weeks due to bone marrow failure, severe infections, or bleeding (3, 4). Notably, over 50% of ALL cases occur in children. While the cure rate for childhood ALL (cALL) has increased to 80–90% in recent years, several high-risk subtypes still have high relapse rates and poor treatment results, which poses a serious risk to pediatric survival (5–8). Therefore, it is still crucial to conduct continuous research into novel therapeutic approaches in order to enhance clinical results for these individuals.

Ubiquitination plays a crucial role in various biological functions, such as regulating signaling pathways, maintaining cellular homeostasis, and degrading proteins (9–11). Recent studies have highlighted its significant impact on the initiation, progression, and treatment resistance of ALL. For instance, ubiquitination regulates the degradation of Notch1, ensuring the stability of the Notch signaling pathway and preventing T-ALL development (12, 13). However, mutations in the E3 ubiquitin ligase FBW7 can disrupt this balance, triggering the onset of ALL (14–16). Moreover, ubiquitination targets extend to cell cycle regulators, apoptosis-related proteins, and immune checkpoint molecules. Evidence indicates that MDM2-mediated accelerated degradation of p53 and abnormal ubiquitination of key cell cycle proteins, such as Cyclins and CDKs, contribute to the uncontrolled proliferation and survival of ALL cells (17, 18). Similarly, dysregulated degradation of PD-L1 can impair the efficacy of CAR-T cell therapy and targeted immunotherapy (19).

Emerging evidence underscores the therapeutic potential of targeting ubiquitination pathways in ALL. For example, proteasome inhibitors like bortezomib have shown efficacy in newly diagnosed T-ALL (20). Additionally, it has been reported that E3 ubiquitin ligase FBW7 is linked to NOTCH1-driven T-ALL (21). Recent study also reveal that ubiquitination of immune checkpoints (e.g., PD-L1) regulates their stability, offering a dual therapeutic strategy to enhance immunotherapy efficacy (22). Furthermore, novel inhibitors targeting deubiquitinating enzymes such as USP7 or USP14 are under investigation for their ability to restore tumor suppressor functions and overcome chemoresistance (23). Despite these advances, the heterogeneity of ubiquitination-related mechanisms in ALL remains underexplored. Comprehensive profiling of ubiquitination-related genes (URGs) could therefore uncover subtype-specific vulnerabilities and guide the development of precision therapies.

Our study aimed to find the important part ubiquitination plays in ALL and the necessity for future research to provide more accurate and potent treatment plans. These findings not only advance our understanding of ubiquitination in ALL but also highlight actionable targets for improving treatment outcomes, particularly in high-risk patients who face limited therapeutic options. Pediatric patients and those with recurrent disease may benefit greatly from such developments.

## 2 Methods

### 2.1 Data acquisition

The transcriptome sequencing data for ALL participants were collected from the “Therapeutically Applicable Research to Generate Effective Therapies” (TARGET) database. Following the exclusion of samples with missing clinical information or survival lengths of less than 30 days, the analysis comprised 464 cALL patients with complete mRNA sequencing data and extensive clinical information. Ubiquitination-related genes (URGs) were found by thoroughly reviewing pertinent datasets from the GSEA database (https://www.gsea-msigdb.org/gsea/index.jsp) and the Genecards database (https://www.genecards.org/). A total of 1121 URGs with strong research evidence were curated and confirmed for further study (**Supplementary Table 1**).

### 2.2 Consensus clustering analysis

Consensus clustering was performed in R (version 4.1.0) with the ConsensusClusterPlus package to identify molecular subgroups of ALL patients based on URG expression profiles. Resampling was performed 1,000 times with a subsample ratio of 0.8, and the best number of clusters (k = 4) was identified using the consensus cumulative distribution function (CDF) and delta area plot. Patients were divided into four subtypes: clusters A, B, C, and D.

Principal Component Analysis (PCA), done with the factoextra package, was used to confirm and illustrate the clustering results, emphasizing the significant separation of subtypes. Heatmaps created with the ComplexHeatmap package showed the expression patterns of URGs across clusters, supporting the robustness of the discovered molecular subtypes. Survival Analysis Kaplan-Meier survival curves were created to assess the overall survival (OS) of patients in various clusters or risk groups. The analysis was carried out in R, with the survival package calculating survival probability and the survminer package visualizing the Kaplan-Meier curves. Log-rank tests were used to determine statistical significance and were accomplished using the survdiff function in the survival package.

### 2.3 Differential Gene Expression Analysis

Based on the subtypes discovered using consensus clustering and the findings of survival analysis, Cluster D was designated as the high-risk category with the worse prognosis among ALL patients. Using R’s limma package, we discovered differentially expressed genes (DEGs) between Cluster D and the other clusters. DEGs were chosen based on an adjusted p-value < 0.05 and |log2 fold-change| > 0.585, indicating genes strongly related with the high-risk condition.

### 2.4 Construction of risk score (prognosis) model

To identify ubiquitination-related genes (URGs) that are significantly associated with prognosis and develop a prognostic prediction model, 69 genes that overlap with URGs and differentially expressed genes (DEGs) were subjected to LASSO regression, as well as univariate and multivariate Cox regression analyses. These studies were carried out in R with the glmnet package for LASSO regression and the survival package for Cox regression.

The dataset was randomly divided into two sets: training and validation, at a 1:1 ratio. Based on the Cox regression results, a risk score formula was created by adding the expression levels of the final risk-associated genes, which were weighted by their respective Coef coefficients. The coefficient of the final 9-gene signature formula was displayed in **Supplementary Table 3**. Each patient sample received a risk score, and all samples were divided into high-risk and low-risk groups using the median risk score as the cutoff. This model was validated as an effective predictor of patient prognosis using Receiver Operating Characteristic (ROC) curve analysis with the TimeROC program.

### 2.5 Functional enrichment analysis

Using the same screening criteria, we discovered risk-related differentially expressed genes (RDEGs) between the high-risk and low-risk groups. The differential analysis was performed using the wilcox test, with genes having a log fold change (logFC) greater than 0.585 and a p-value less than 0.05 defined as RDEGs. Functional enrichment analysis, which included Gene Ontology (GO) and KEGG pathway analyses, was used to investigate the biological processes, cellular components, and molecular functions associated with these RDEGs. The enrichment analysis and visualization were carried out in R with the clusterProfiler tool. GO analysis revealed critical biological processes, cellular components, and molecular functions, whereas KEGG pathway analysis showed relevant signaling and metabolic pathways related with the RDEGs.

### 2.6 Immune landscape analysis

Previous research has shown that the tumor microenvironment (TME) is crucial for tumor progression (24, 25). In this study, the CIBERSORT algorithm was used to compare the makeup of immune cells in high-risk and low-risk ALL patients. Data visualization was carried out in R using the ggplot2 and ComplexHeatmap packages to show significant variations in immune cell distribution between the two risk groups (p-values computed using the Wilcox test).

Single-sample Gene Set Enrichment Analysis (ssGSEA) with the GSVA program was used to determine key immunological characteristic scores such as antigen presentation capacity, inflammatory activity, and cytotoxicity (26) Furthermore, the expression levels of classical immune checkpoint genes (e.g., PDCD1, CTLA4, LAG3) were compared between high-risk and low-risk groups to better understand the function of immune regulation in ALL progression.

The “pRRophetic” R software was used to perform drug sensitivity analysis, which estimates IC50 values for various medicines based on gene expression profiles and data from the GDSC database. The Wilcoxon rank-sum test was used to analyze statistical differences in drug sensitivity between high-risk and low-risk ALL groups. The results, displayed as boxplots, revealed medicines with significantly varying IC50 values, indicating prospective options for personalized therapy regimens in ALL.

### 2.7 Cell lines and culture conditions

Jurkat and 293T cells were cultured in RPMI-1640 medium (Thermo Fisher Scientific) and Dulbecco’s Modified Eagle’s Medium (DMEM; Thermo Fisher Scientific), which were both supplemented with 10% fetal bovine serum (FBS; Gibco) and 1% penicillin-streptomycin. To reduce FBXO8 expression, a particular short hairpin RNA (shRNA) sequence was developed and cloned into the pLKO.1 vector (Plasmid #10879) with EcoRI and AgeI restriction enzymes (New England Biolabs). Lentiviral transduction was carried out using the packaging plasmids psPAX2 (Plasmid #12260) and pMD2.G (Plasmid #12259). Cells were centrifuged at 1,000 rpm for 1.5 hours at 37°C and transduced with 8 µg/ml polybrene (Sigma-Aldrich). Transduced cells were chosen for 48 hours with 1 µg/ml puromycin (Beyotime Biotechnology), and knockdown effectiveness was verified by qPCR (Thermo Fisher Scientific). The primer sequences for FBXO8 used in the *in vitro* investigation were as follows: Forward: CCGGTATGACAACATCTACCTTATTCTCGAGAATAAGGTAGATGTTGTCATATTTTTG. Reverse: AATTCAAAAATATGACAACATCTACCTTATTCTCGAGAATAAGGTAGATGTTGTCATA.

### 2.8 qPCR analysis

Total RNA was isolated from cells using the TRIzol reagent (Thermo Fisher Scientific) per the manufacturer’s instructions. cDNA was generated from 1 µg of total RNA using the High-Capacity cDNA Reverse Transcription Kit (Thermo Fisher Scientific). SYBR Green Master Mix (Thermo Fisher Scientific) was used to run qPCR on an Applied Biosystems QuantStudio 5 Real-Time PCR System. The FBXO8 expression was normalized to GAPDH, and relative expression levels were estimated using the 2^(-ΔΔCt) technique. **Supplementary Table 3** contains primer sequences for *FBXO8* and its negative control, GAPDH. Each sample was examined three times to verify repeatability.

### 2.9 Apoptosis analysis by flow cytometry

Apoptosis was detected with the Annexin V-FITC/PI Apoptosis Detection Kit (BD Biosciences). The cells were extracted, washed twice with cold PBS, and resuspended in binding buffer at a concentration of 1 × 10^6 cells/ml. Next, add 5 µl of Annexin V-FITC and 5 µl of propidium iodide (PI) to 100 µl of cell suspension. The samples were incubated in the dark at room temperature for 15 minutes before being examined using a BD LSRFortessa flow cytometer (BD Biosciences).

### 2.10 T-ALL mouse model construction

T-ALL models were established using C57BL/6J mice aged 8 to 12 weeks. Retroviruses carrying the MSCV-NOTCH1-IRES-GFP sequence were generated by transfecting 293T cells with plasmids using polyethylenimine (PEI; Polysciences). Viral supernatants were collected at 48 and 72 hours post-transfection. Bone marrow (BM) cells were harvested from mice pre-treated with 5-fluorouracil (5-FU; Sigma-Aldrich) to enrich hematopoietic stem and progenitor cells (HSPCs). These BM cells were infected with the retroviruses in the presence of polybrene (8 µg/ml; Sigma-Aldrich) and cultured in Iscove’s Modified Dulbecco’s Medium (IMDM; Thermo Fisher Scientific) supplemented with 10% fetal bovine serum (FBS; Gibco) and 1% penicillin-streptomycin (Thermo Fisher Scientific). Infected cells were transplanted intravenously into lethally irradiated (9 Gy) recipient mice (X-RAD 320; Precision X-Ray).

*FBXO8* knockdown was achieved by lentiviral transduction using vectors carrying FBXO8-specific shRNA, packaged with psPAX2 and pMD2.G plasmids. The shRNA sequence for FBXO8 was as follows: AATTAAAAGAGAATCTATCTTGATGAAAGTTCGCTTTCATCAAGATAGATTCTC; Reverse: CCGGGAGAATCTATCTTGATGAAAGCGAACTTTCATCAAGATAGATTCTCTTTT. The scramble shRNA sequence was set as negative control (Plasmid # 1864). Lentiviral particles were produced in 293T cells and transduction efficiency was enhanced with polybrene. Knockdown was confirmed by qPCR using SYBR Green Master Mix (Thermo Fisher Scientific).

### 2.11 Leukemic cell transplantation

Primary leukemic cells were cultured in RPMI-1640 medium (Thermo Fisher Scientific) supplemented with 10% FBS, SCF (PeproTech), IL-3 (PeproTech), IL-6 (PeproTech), and GM-CSF (PeproTech), all at 10 ng/ml. The cells were infected with retroviruses, centrifuged (1,500 rpm, 1.5 hours; Eppendorf 5804R), and cultured for 2 hours before the media was replaced. Puromycin (2 µg/ml; Beyotime) was used to select infected cells over two days. Leukemic cells from T-ALL mice were administered intravenously into mice that had been sub-lethally irradiated (4.5 Gy).

Survival, weight changes, and leukemic progression were all observed in recipient mice. The leukemic load in BM and peripheral blood was determined using flow cytometry (BD LSRFortessa). All animal research followed ethical rules established by the Ruiye Model Animal (Guangzhou) Biotechnology Co., Ltd. Laboratory Animal Ethics Committee (RYEth-20240329576).

## 3 Results

### 3.1 Molecular subtypes of ALL identified by consensus clustering

**Figure 1:**
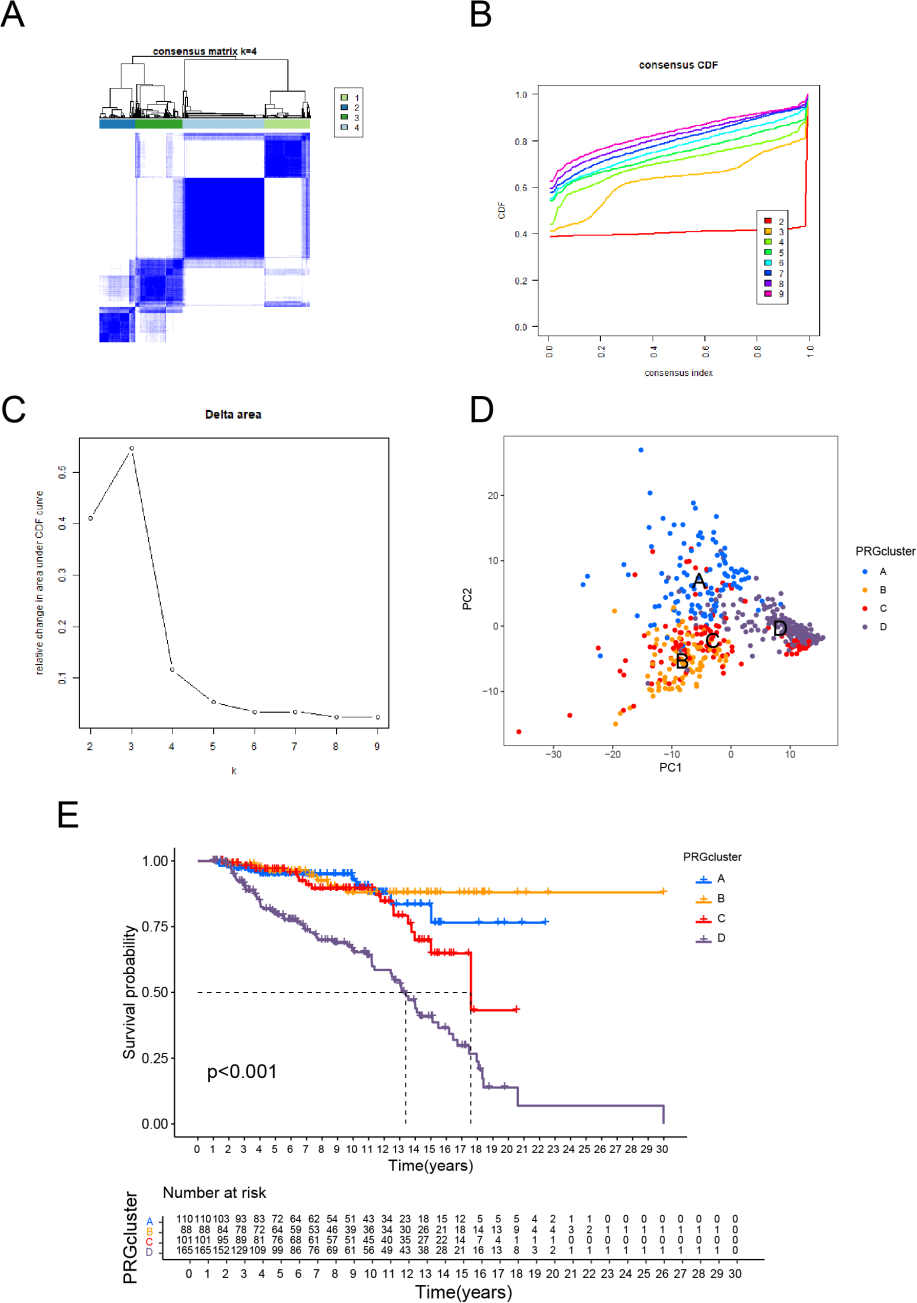
Molecular Subtypes of ALL Identified by Consensus Clustering and Their Prognostic Implications. **(A)** Consensus clustering matrix for k = 4, showing clear separation of the four clusters. **(B)** Consensus cumulative distribution function (CDF) plot for different cluster numbers, indicating stable clustering results. **(C)** Delta area plot showing the relative change in the area under the CDF curve, with k = 4 selected as the optimal number of clusters. **(D)** PCA illustrating the distribution of samples across the four identified clusters. **(E)** Kaplan-Meier survival curves showing significant differences in overall survival among the four clusters.

Based on consensus clustering analysis, ALL patients were categorized into four distinct molecular subtypes (Clusters A, B, C, and D; **Figures 1A–C**). PCA revealed clear separation of the four subtypes in two-dimensional space, reflecting significant differences in gene expression profiles among them (**Figure 1D**). This robust classification provides a solid foundation for subsequent investigations into biological functions.

Kaplan-Meier survival analysis revealed substantial variations among subtypes, with Cluster D having the lowest overall survival and Cluster B having the best prognosis (p < 0.001, **Figure 1E**). These findings reveal specific biological factors that drive the progression and prognosis of each subtype. The PCA and survival analysis results are consistent, which verifies the subtype classification’s dependability. Cluster D’s much lower survival rates as a high-risk subtype indicate the presence of distinct molecular processes connected to malignancy, necessitating additional exploration.

### 3.2 Construction and evaluation of risk prognosis model

**Figure 2:**
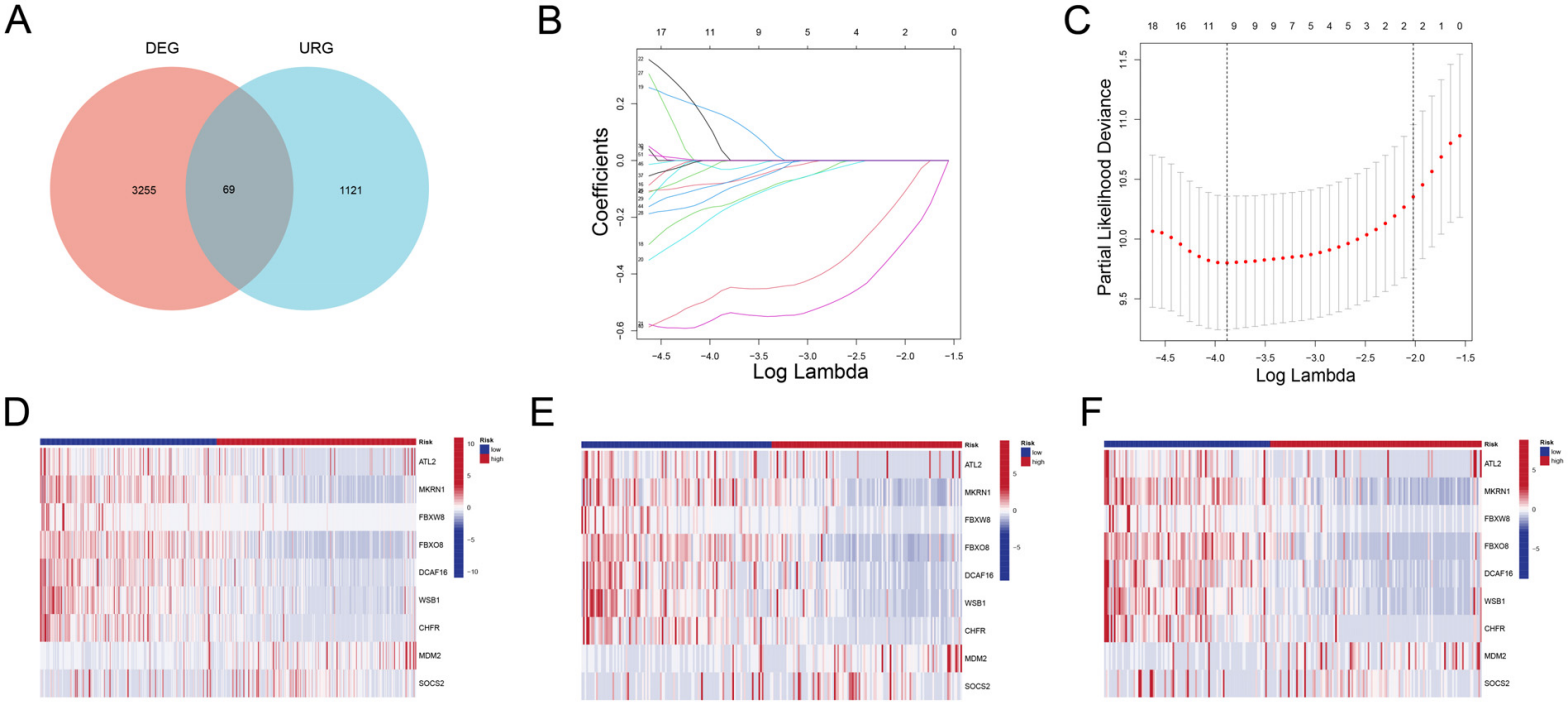
Identification of Prognostic Genes and Expression Patterns **(A)** Venn diagram showing 67 overlapping genes between DEGs and URGs. **(B)** LASSO coefficient profiles of the candidate genes. **(C)** Partial likelihood deviance plot used to determine the optimal lambda value, selecting nine key genes. **(D-F)** Heatmaps showing the expression patterns of the nine key genes in high-risk and low-risk groups for all samples **(D)**, training set **(E)**, and validation set **(F)**. High-risk patients exhibit significantly different expression profiles compared to low-risk patients.

Using a Venn diagram (**Figure 2A**), 69 potential prognostic genes were discovered. These were refined further using LASSO regression analysis (**Figures 2B, C**), resulting in the identification of nine key genes (*ATL2*, *MKRN1*, *FBXW8*, *FBXO8*, *DCAF16*, *WSB1*, *CHFR*, *MDM2*, *SOCS2*) that were found to be strongly associated with the prognosis of ALL patients, as determined by univariate (**Supplementary Figure 1**) and multivariate COX regression analyses. The expression heatmaps (**Figures 2D-F**) show that there are significant changes in the distribution of these important genes between high-risk and low-risk patient groups across all data, including the training and validation sets.

**Figure 3:**
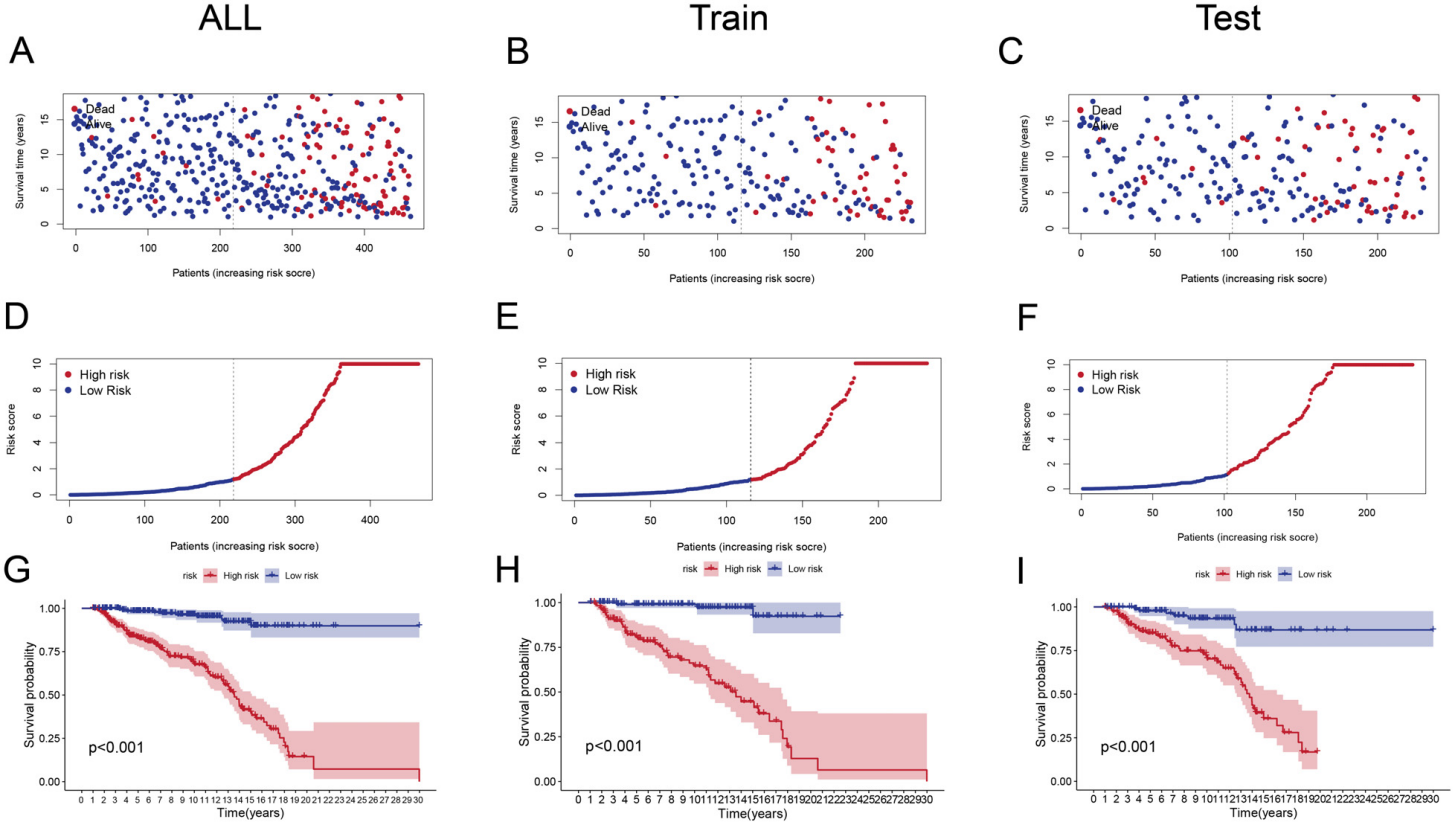
Risk Score Distribution and Survival Analysis **(A–C)** Scatter plots showing survival status (alive or dead) of ALL patients in the entire cohort **(A)**, training set **(B)**, and test set **(C)** ranked by increasing risk scores.**(D–F)** Distribution of risk scores in high-risk and low-risk groups for the entire cohort **(D)**, training set **(E)**, and test set **(F)**. High-risk groups have significantly higher scores. **(G-I)** Kaplan-Meier survival curves for the entire cohort **(G)**, training set **(H)**, and test set **(I)**, illustrating significantly poorer survival outcomes in the high-risk group (*p* < 0.001).

Patients in the high-risk group had significantly lower survival durations than those in the low-risk group (p < 0.001) across the overall cohort (**Figures 3A, D, G**), training (**Figures 3B, E, H**), and test sets (**Figures 3C, F, I**). The Kaplan-Meier survival curves supported the risk score’s strong prediction ability for the ALL prognosis.

**Figure 4:**
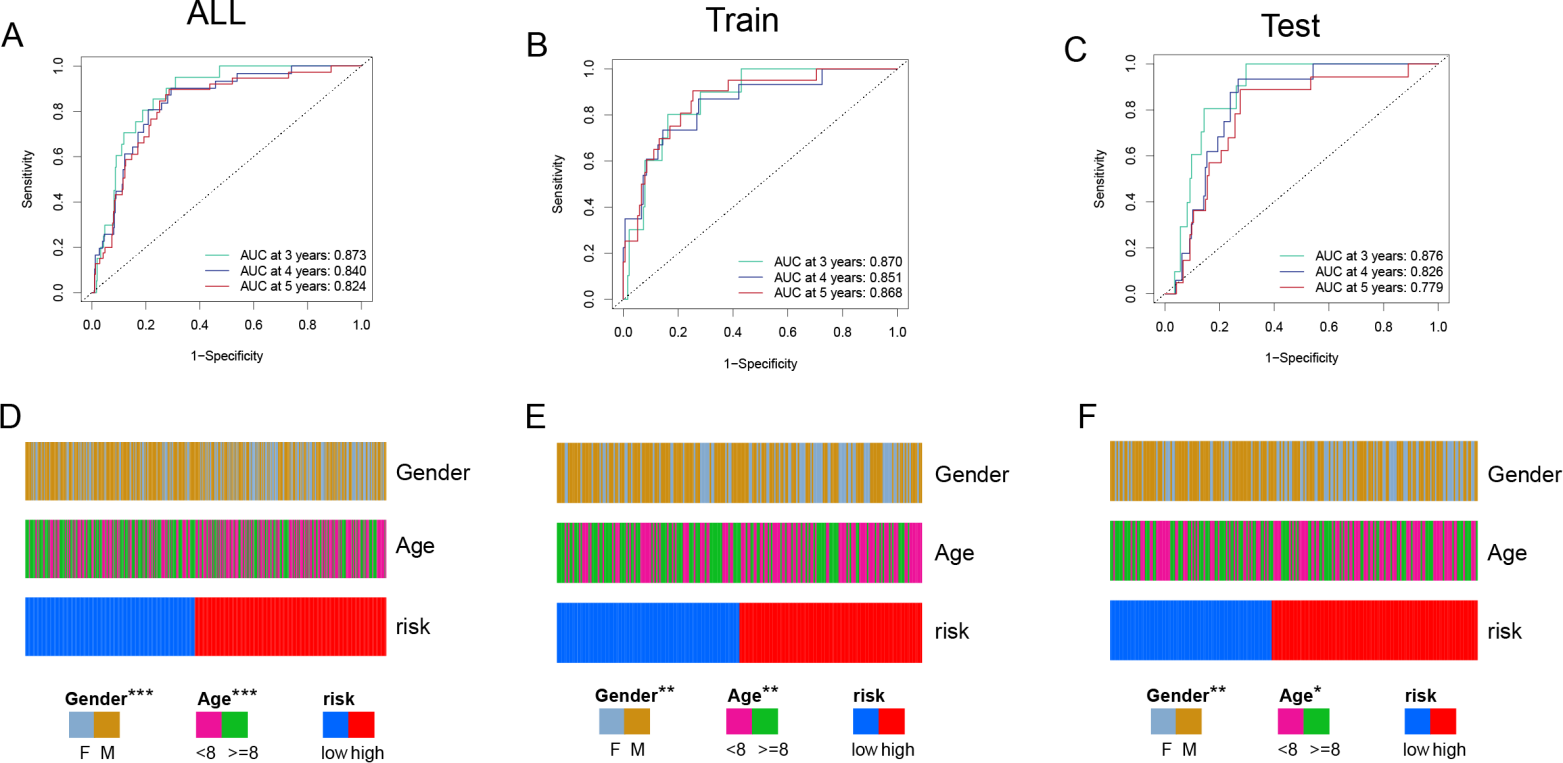
Model Performance and Clinical Association Analysis **(A, C)** ROC curves evaluating the predictive performance of the risk model in all samples **(A)**, training set **(B)**, and test set **(C)**. The AUC values at 3, 4, and 5 years demonstrate the model's strong predictive ability. **(D-F)** Clinical characteristic distribution, showing the association of gender, age, and risk groups in all samples **(D)**, training set **(E)**, and test set **(F)**. Significant correlations between risk scores, gender, and age are observed. Chi-squared test was employed. (*p*<0.05*, *p*<0.01**, *p*<0.001***).

ROC curve analysis (**Figures 4A-C**) was used to evaluate the model’s predictive performance across 3, 4, and 5 years. The AUC values for the total cohort were 0.873, 0.840, and 0.824; for the training set, 0.870, 0.851, and 0.868; and for the test set, 0.876, 0.826, and 0.779, all indicating good discrimination ability. Furthermore, incorporating clinical factors into the study (**Figures 4D-F**) indicated substantial relationships between the risk score and patient gender and age, demonstrating the model’s clinical significance.

### 3.3 Functional enrichment analysis

**Figure 5:**
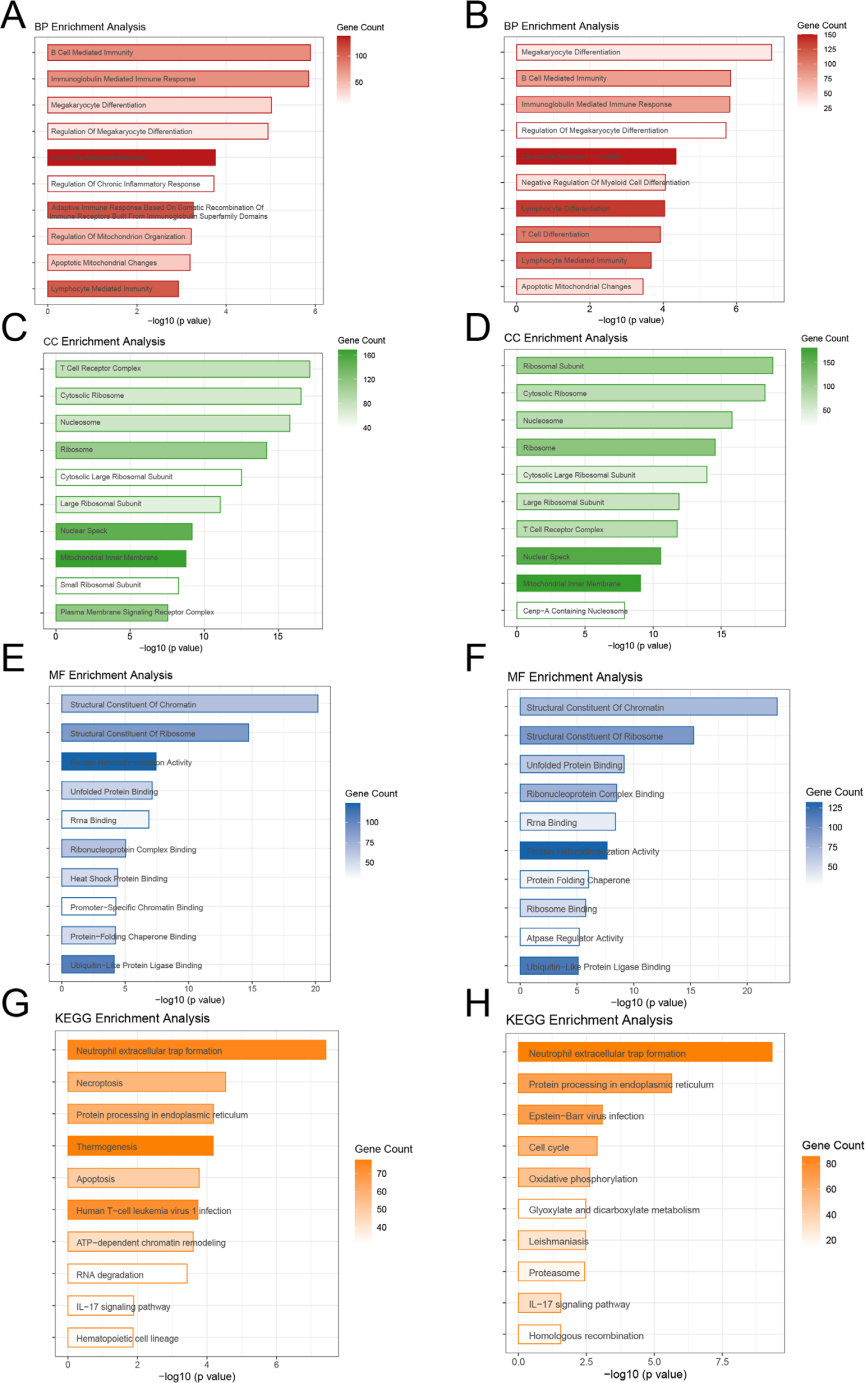
Functional Enrichment Analysis of High-Risk and Low-Risk Groups **(A, B)** Biological processes showing immune regulation and differentiation as key features in both cohorts. **(C, D)** Cellular components enriched in nucleosomes, nucleoli, and mitochondrial structures.**(E, F)** Molecular functions highlighting chromatin and ribosome-related activities. **(G, H)** KEGG pathways showing inflammation and apoptosis dominance in the training cohort, and metabolism and proliferation in the validation cohort.

The functional enrichment analysis indicated significant differences between the high-risk and low-risk groups in biological processes, cellular components, molecular activities, and pathways. In terms of biological processes, the training-cohort was significantly enriched in immune regulation-related processes such as B cell-mediated immunity, immunoglobulin-mediated immune response, megakaryocyte differentiation, and lymphocyte-mediated immunity (**Figure 5A**), whereas the validation cohort was enriched in megakaryocyte differentiation, T cell differentiation, lymphocyte immunity, and mitochondrial apoptosis-related processes (**Figure 5B**). In terms of cellular components, both cohorts showed significant enrichment in key structures such as nucleosomes, nucleoli, and mitochondrial inner membranes (**Figures 5C, D**); In molecular function analysis, the DEGs in training cohort was more enriched in chromatin structural components, ribosome structural components, and unfolded protein binding (**Figures 5E, F**), whereas the DEGs in validation cohort was also significantly associated with ribosome binding and ATPase regulatory activity. Pathway analysis revealed that the high-risk group was considerably enriched in inflammation and apoptosis-related pathways, such as neutrophil extracellular trapping formation (NETosis), apoptosis, protein processing in the endoplasmic reticulum, and the IL-17 signaling pathway. In contrast, the validation cohort had higher levels of proliferation and metabolism-related pathways, such as the cell cycle, oxidative phosphorylation, glycolysis, and Epstein-Barr virus infection (**Figures 5G, H**). Overall, the DEGs in training cohort was more associated with inflammation, immune response, and apoptosis, whereas the DEGs in validation cohort was primarily involved in metabolism and proliferation-related processes, providing important insights for future research into the molecular mechanisms of ALL and precision treatment strategies.

### 3.4 Immune landscape analysis

**Figure 6:**
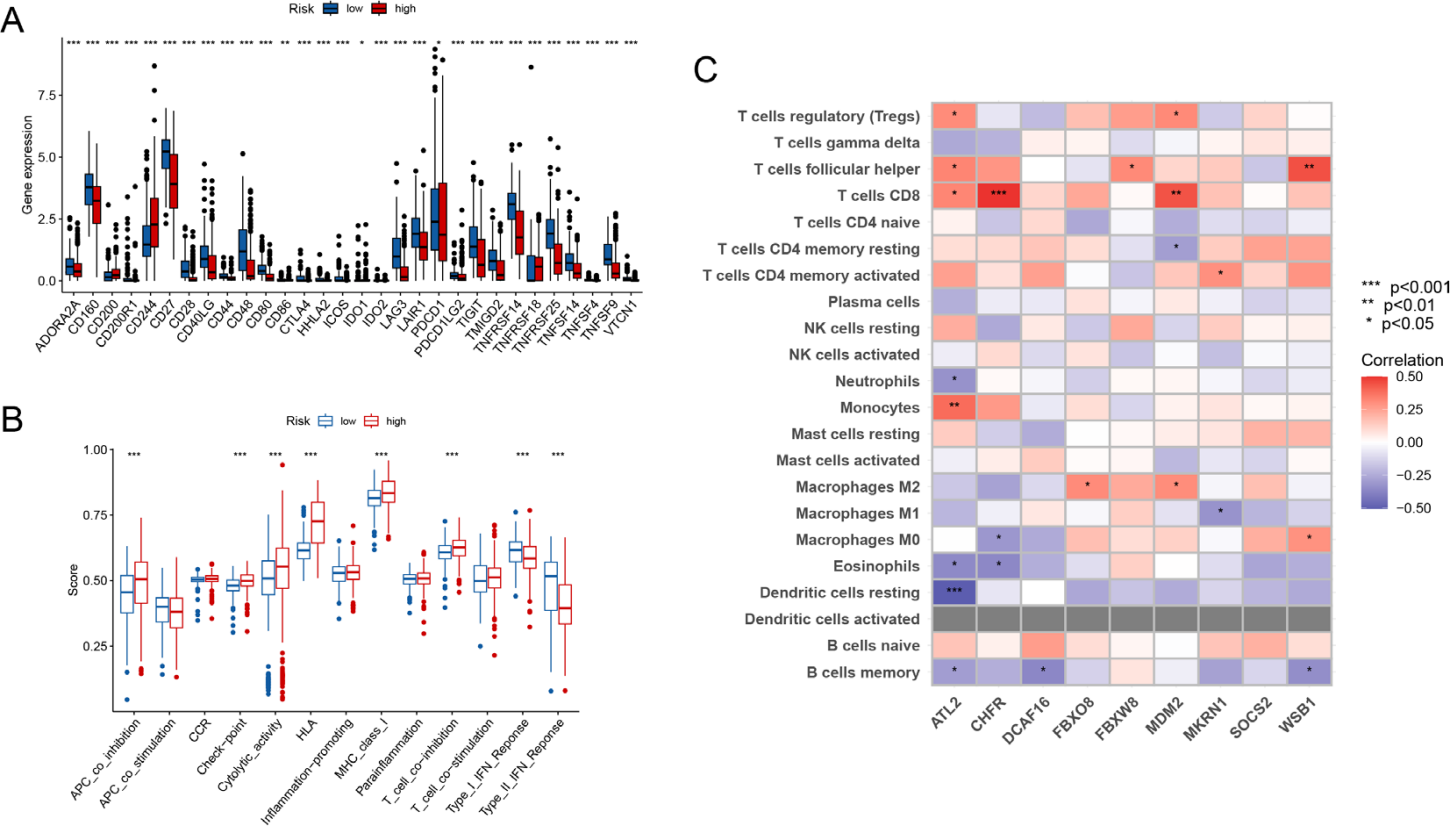
Immune Landscape Analysis **(A)** Elevated immune checkpoint genes (e.g., PDCD1) in the high-risk group compared to the low-risk group. **(B)** Immune feature scores show significant differences in antigen presentation and T cell function between groups. **(C)** Immune cell infiltration was linked with important gene expression. (*p*<0.05*, *p*<0.01**, *p*<0.001***).

Immune checkpoint gene expression levels differed significantly between high-risk and low-risk groups. Patients in the high-risk group had significantly higher expression of immune checkpoint genes such as *PDCD1(PD-1)*, whereas certain immune-related genes were overexpressed in the low-risk group. These results indicate that the high-risk group may have a more active immunological escape mechanism (**Figure 6A**). Furthermore, the immunological feature scores differed significantly between the two groups. The high-risk group scored significantly higher on antigen presentation inhibition (APC co-inhibition), inflammatory activity, and T cell co-stimulation, while the low-risk group scored higher on antigen presentation activation (APC co-stimulation) (**Figure 6B**).

Immune cell infiltration was also found to be substantially linked with important gene expression (**Figure 6C**). Treg cells and M2 macrophages in the high-risk group correlated positively with important genes including *FBXO8* and *MDM2*. In contrast, activated CD4+ memory T cells were positively related to the expression of *MKRN1*. These data highlight that the high-risk group has an immunosuppressive milieu, whereas the low-risk group is more related with an immune-activated state.

### 3.5 Drug sensitivity analysis

**Figure 7:**
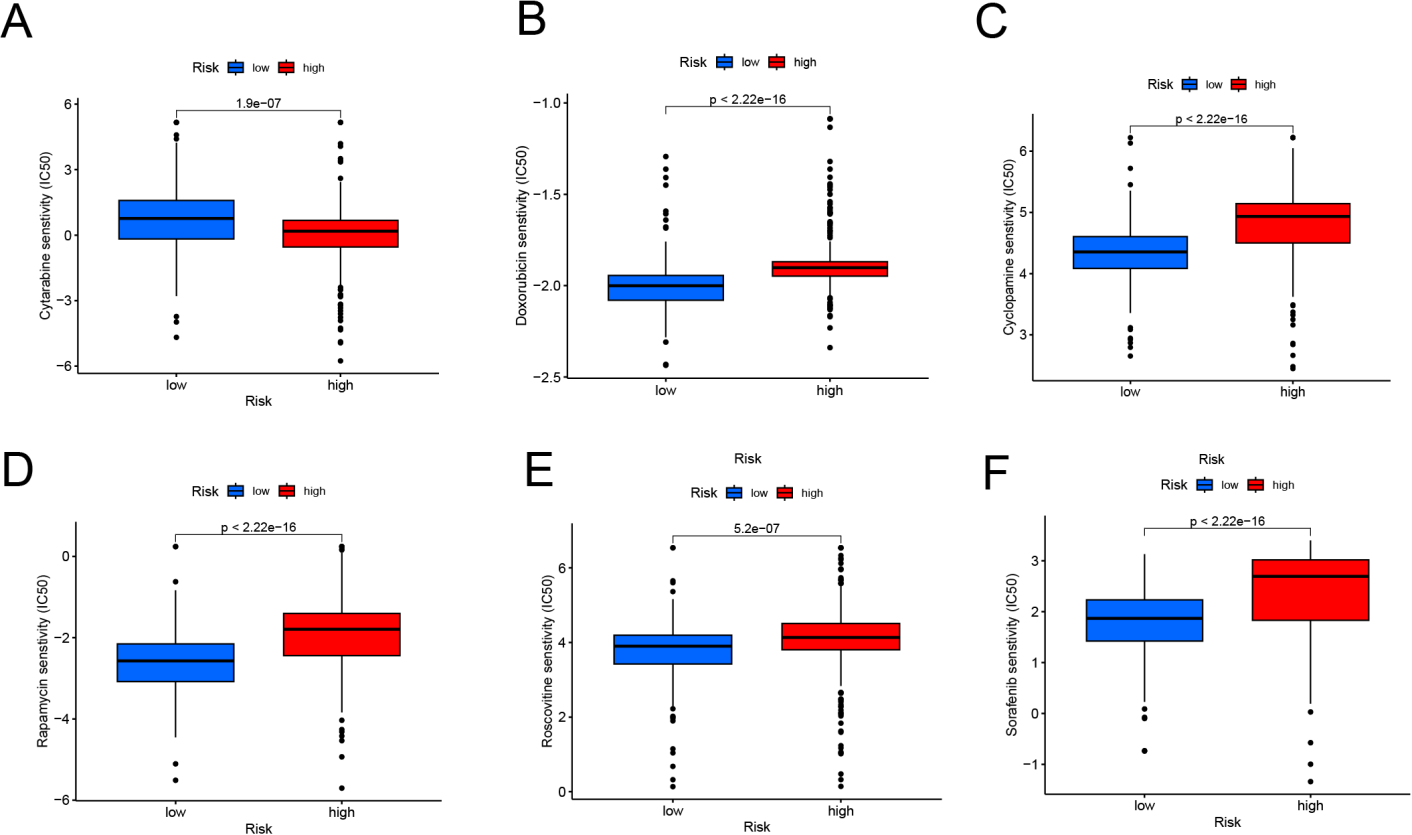
Drug Sensitivity Analysis in High-Risk and Low-Risk Groups **(A, F)** Boxplots showing predicted IC50 values for cytarabine **(A)**, doxorubicin **(B)**, cyclopamine **(C)**, rapamycin **(D)**, roscovitine **(E)**, and sorafenib **(F)**. The high-risk group demonstrates significantly higher IC50 values for most drugs, indicating reduced drug sensitivity compared to the low-risk group (*p* < 0.001).

The study found significant differences in medication sensitivity between the high-risk and low-risk groups. The high-risk group had higher sensitivity to routinely used medications such as cytarabine (**Figure 7A**, p = 1.9e-07), targeted medicines such as doxorubicin (**Figure 7B**, p < 2.22e-16), cyclopamine (**Figure 7C**, p < 2.22e-16). rapamycin (**Figure 7D**, p < 2.22e-16), roscovitine (**Figure 7E**, p = 5.2e-07), and sorafenib (**Figure 7F**, p < 2.22e-16) demonstrated lower sensitivity in the high-risk group. These findings show considerable medication resistance in the high-risk population, which may contribute to their poor prognosis and pose possible hurdles for treatment management.

### 3.6 FBXO8 knockdown inhibits apoptosis and promotes ALL progression

**Figure 8:**
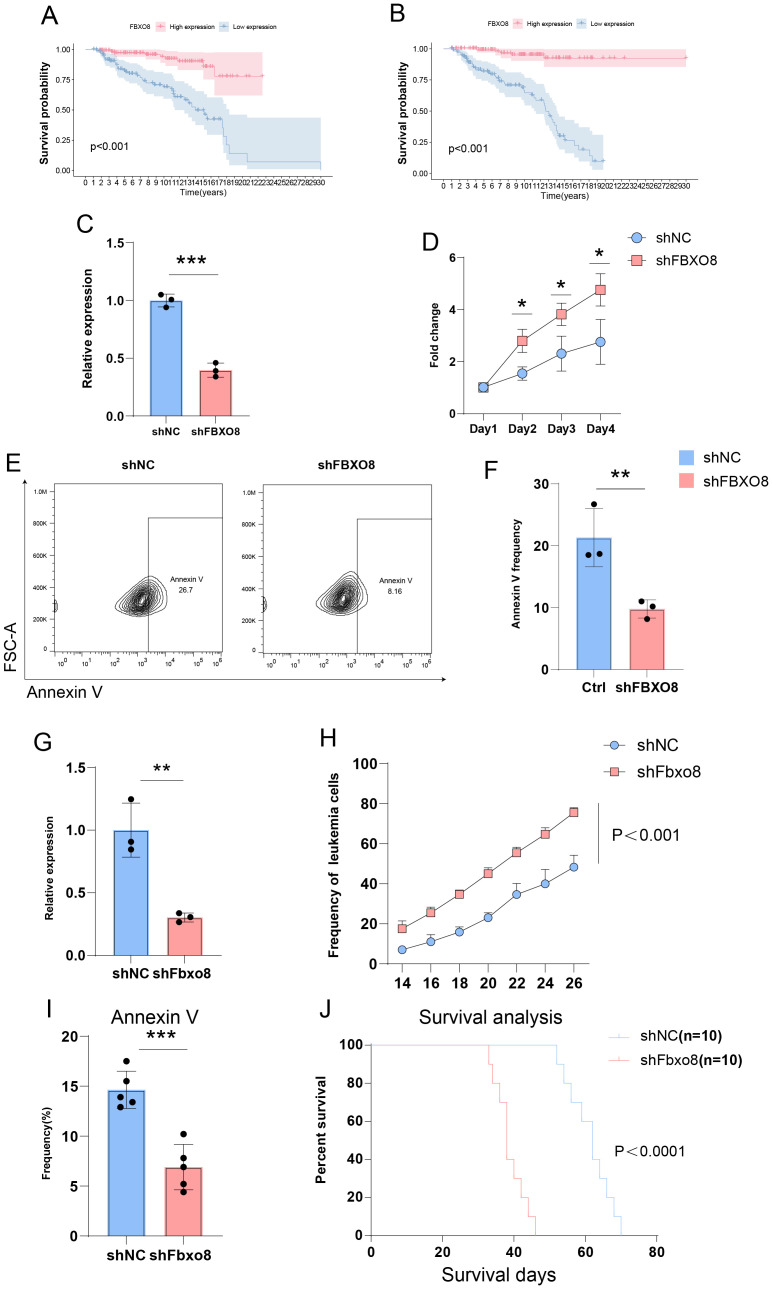
Impact of FBXO8 Knockdown on ALL Progression **(A, B)** Kaplan-Meier survival curves showing significantly better survival in patients with high FBXO8 expression (*p* < 0.001). **(C–F)** Cellular experiments demonstrating reduced FBXO8 expression after knockdown **(C)**, promotion of proliferation **(D)**, and suppression of apoptosis as indicated by Annexin V-positive cells **(E, F)**. **(G-J)** Animal experiments confirming reduced FBXO8 expression **(G)**, enhanced tumor growth **(H)**, inhibited apoptosis **(I)**, and lower survival rates **(J)** in the shFBXO8 group (*p*<0.05*, *p*<0.01**, *p*<0.001***).

To discover possible treatment targets, we ran survival analyses on the nine main genes in the prognostic model. Kaplan-Meier survival curve analysis showed that ALL patients with high *FBXO8* expression had significantly improved survival rates, whereas those with low *FBXO8* expression had significantly worse outcomes, both in the training and validation cohorts (**Figures 8A, B**, p < 0.001). qPCR analysis showed that sh*FBXO8* therapy dramatically reduced *FBXO8* expression relative to the shNC control group (**Figure 8C**, p < 0.001). In cell proliferation experiments, the sh*FBXO8* group’s proliferation rate was significantly higher than the control group (**Figure 8D**, p < 0.001). Flow cytometry analysis showed a significant decrease in Annexin V-positive cells after sh*FBXO8* treatment. (**Figures 8E, F**, p < 0.01). This suggests that *FBXO8* knockdown decreases apoptosis in ALL cells.

Given the variations in T cell invasion and function shown by the immune landscape study, we focused on developing a T-ALL mice model for further investigation. *FBXO8* expression decreased significantly after knockdown (**Figure 8G**, p < 0.01). Tumor burden tests demonstrated that the sh*Fbxo8* group exhibited a notably faster growth of GFP+ leukemia cells compared to the control group (**Figure 8H**, p < 0.001). Flow cytometry showed a significant decrease in Annexin V-positive cells in the sh*FBXO8* group (**Figure 8I**, p < 0.001), indicating that *FBXO8* knockdown inhibits apoptosis *in vivo*. Furthermore, Kaplan-Meier survival analysis revealed that mice in the sh*FBXO8* group had a considerably reduced survival rate compared to controls (**Figure 8J**, p < 0.0001), highlighting the crucial function of *FBXO8* in ALL progression.

## 4 Discussion

Although the overall cure rate of cALL is high, high-risk subtypes face limited treatment efficacy and high recurrence rates, with treatment resistance and long-term toxicity remaining pressing clinical challenges (27, 28). Extensive studies have demonstrated the critical and multifaceted role of ubiquitination in ALL. Comprehensive research into the ubiquitination landscape in ALL can offer valuable insights into tumorigenesis mechanisms and guide the development of precision therapies (29–31). Our study integrated ubiquitination-related genes with transcriptome sequencing data of ALL from the TARGET dataset, identifying four distinct ubiquitination subtypes through consensus clustering. Using methods such as LASSO and COX regression, we constructed a prognostic prediction model based on nine ubiquitination-related genes, which effectively stratifies patients by risk and predicts their prognosis. Among these genes, *FBXO8* emerged as the most promising target for ubiquitination-related regulatory intervention in ALL. Its role in ALL progression was further validated through *in vivo* and *in vitro* experiments.

Our study highlights significant associations between patient age, gender, and prognosis in ALL. Age is a well-established prognostic factor in ALL, with pediatric patients generally exhibiting different prognosis compared to adolescents and adults. Pediatric ALL is frequently associated with favorable genetic alterations, such as ETV6-RUNX1 fusion or hyperdiploidy, which confer sensitivity to chemotherapy (28). In contrast, adult ALL is enriched in high-risk genetic subtypes (e.g., BCR-ABL1 or KMT2A rearrangements) and somatic mutations (e.g., TP53), which drive chemoresistance and relapse (28, 32). Additionally, pediatric patients typically retain robust immune surveillance, characterized by higher cytotoxic T cell activity and lower immunosuppressive cell infiltration (24). Gender may also have impact on prognosis of cALL patients. Females exhibit bi-allelic expression of X-chromosome genes, which may buffer against mutations in critical tumor suppressors (e.g., UTX) located on the X chromosome (33).

In this study, we constructed a prognostic model based on nine key URGs (*ATL2*, *MKRN1*, *FBXW8*, *FBXO8*, *DCAF16*, *WSB1*, *CHFR*, *MDM2*, and *SOCS2*), providing new insights into the molecular mechanisms underlying ALL progression. Among the nine genes identified, several have been implicated in cancer development and progression through their involvement in protein ubiquitination and degradation. *ATL2* dysfunction may impact ER stress responses, which may link to leukemogenesis and drug resistance in leukemia cells (34). It has been reported that *MKRN1* contains a functional ring-finger structural domain of E3 ubiquitin ligase which may activate TGF-β signaling pathway to promote colorectal cancer metastasis (35). Paradoxically, our analysis identified MKRN1 as a risk-reducing factor. In ALL, *MKRN1* may predominantly act as an RNA stabilizer for pro-survival transcripts, warranting mechanistic validation. *FBXW8* has been shown to be able to bind cullin protein 7 (CUL7), which exerts both tumor promotion and suppression in a context-dependent manner (36). DCAF16, a substrate receptor for CRL4 ubiquitin ligases, mediate degradation of DNA repair proteins (37). Low *DCAF16* expression level may impair genomic stability in ALL patients. WSB1, an E3 ligase component, promotes hypoxia-inducible factor 1α (HIF-1α) degradation under normoxia, which promotes cancer invasion and metastasis (38). CHFR (Checkpoint with Forkhead-associated and RING finger domains) plays a critical role in regulating mitotic entry and implicated in wide range of cancer (39). While *MDM2* overexpression typically promotes p53 degradation and oncogenesis, recent studies reveal context-dependent roles: in p53-mutant ALL, MDM2 may instead degrade pro-survival proteins like NF-κB or stabilize p73, an apoptotic effector (17). SOCS2 (Suppressor of Cytokine Signaling 2) modulates cytokine signaling and ferroptosis of tumor cells, potentially shaping the ALL tumor microenvironment (40).

FBXO8 (F-box only protein 8) is a member of the F-box protein family and a critical component of the E3 ubiquitin ligase complex. F-box proteins interact with the SCF complex to mediate the ubiquitination of specific protein substrates (41). Although research on FBXO8 in solid tumors and hematological malignancies is still in its early stages, evidence suggests it plays a potential role in tumor initiation and progression. Current studies on *FBXO8* in digestive system tumors indicate its predominantly protective role. Downregulation of *FBXO8* expression has been observed in liver cancer (42), gastric cancer (43), renal cancer (44) and colorectal cancer (45), with its reduced expression closely associated with poor patient prognosis. Mechanistically, one study have identified GSTP1 as a substrate of FBXO8-mediated ubiquitination, which suppresses malignant behaviors in colorectal cancer (46). Additionally, research by F. F. Wang et al. revealed that mTOR protein is another ubiquitination substrate of FBXO8 (47). Regarding upstream regulators, the same research team demonstrated that FBXO8’s function is influenced by non-coding RNA miR-223 (47). Furthermore, Hyun Jung Cho et al. reported that FBXO8 interacts with c-Myc, suggesting that c-Myc expression inhibits *FBXO8* activity, thereby promoting tumor malignancy (48). A study by Xiaohui Zhu et al. proposed a novel tumor-suppressive mechanism for FBXO8, showing that it upregulates epithelial and stem cell markers linked to tumor cell dormancy while downregulating mesenchymal and proliferation markers, thereby promoting metastatic dormancy in colorectal cancer cells (45).

These findings emphasize *FBXO8*’s intricate regulatory network in solid hematological malignancies, as well as its diverse role. Although fewer research have been conducted on different cancers, *FBXO8* generally suppresses tumors through a variety of methods. For example, Hajime Yano et al. discovered that *FBXO8* regulates the ubiquitination and degradation of the GTP-binding protein ARF6, which reduces breast cancer invasiveness. Ying Yu et al. found a link between *FBXO8* expression and both pathological grade and prognosis in low-grade gliomas using extensive clinical specimen analysis. Notably, *FBXO8* has not yet been investigated in ALL. In our study, prognostic analysis using the TARGET database found *FBXO8* as a protective factor in ALL patients. *In vitro* and *in vivo*, shutting down *FBXO8* increased ALL cell proliferation while suppressed apoptosis. These data indicate that targeting *FBXO8* has great promise as a unique treatment strategy for ALL.

Despite the hopeful results, this study has a number of drawbacks. First, while the predictive model and the involvement of *FBXO8* were validated through *in vitro* and *in vivo* investigations, the clinical relevance of these findings needs to be proven in larger, independent patient cohorts. Second, while our experiments focused on *FBXO8* knockdown to elucidate its protective role, the impact of *FBXO8* overexpression on ALL progression was not systematically explored. Third, our functional validations primarily relied on cell lines and murine models. The absence of patient-derived xenograft (PDX) models or primary ALL cell validations may restrict the translational relevance of our findings to human clinical scenarios. Subsequent research should prioritize validating these results in patient-derived samples to enhance therapeutic applicability. Finally, using normoxic culture conditions in *in vitro* research may not fully replicate the hypoxic tumor microenvironment seen in ALL. Future research combining clinical validation, deeper mechanistic investigations, and more physiologically relevant models is required to increase the translational potential of these discoveries.

## 5 Conclusion

This study identifies *FBXO8* as a pivotal prognostic biomarker and therapeutic target in ALL. Knockdown of *FBXO8* promotes tumor progression and inhibits apoptosis, underscoring its potential for therapeutic applications. These findings offer valuable insights into the pathogenesis of high-risk ALL and emphasize the importance of further research into ubiquitination-related mechanisms.

## Data Availability

The original contributions presented in the study are included in the article/**Supplementary Material** Further inquiries can be directed to the corresponding author/s.

## Supplementary material

Supplementary Table 1 Ubiquitination-related genes (URGs) involved in this study.

Supplementary Table 2 The coefficient of final 9-gene signature formula.

Supplementary Table 3 Primer sequences used in qPCR analysis.

Supplementary Figure 1 Univariate Cox analysis results of genes significantly associated with the prognosis of ALL patients.
