# Supplementary material for: Ubiquitination and ALL: Identifying FBXO8 as a prognostic biomarker and therapeutic target
Source: Front Immunol. 2025 May 1;16:1554231. doi: 10.3389/fimmu.2025.1554231 (PMC12078231; doi:10.3389/fimmu.2025.1554231)
Supplement: Supplementary Table 1 — Ubiquitination-related genes (URGs) involved in this study. [file Table1.docx]

| Gene Name |  |  |  |  |
| --- | --- | --- | --- | --- |
| UBA3 | sli-1 | March9 | PUB73 | wdr-23 |
| UBA2 | rnf2 | March5 | UFD2 | DCAF6 |
| SAE1A | trim33 | ORTHL | UBE4B | DCAF12 |
| SAE1B-1 | brd-1 | PELI2 | TUD1 | ERCC8 |
| Sae1 | mib1 | ATL59 | UPS | AMA1 |
| ECR1 | FANCL | TRIM65 | PUB57 | slp1 |
| ATG7 | Bre1 | RHA1B | PUB20 | srw1 |
| UBA1 | sina | At4g11680 | TRAF3IP2 | pop2 |
| ula-1 | Traf6 | RHF1A | PUB14 | ATG16L1 |
| UBA7 | Diap1 | BRCA1 | Os04g0437300 | WDR5 |
| aos-1 | CG11321 | CBLC | PUB12 | WDR61 |
| ULA1 | Pcgf2 | TRIM43 | PUB17 | PAFAH1B1 |
| uba-2 | Pex12 | TRIM38 | PUB45 | TCEB1 |
| rad31 | Roc2 | MYLIP | PUB22 | elc-1 |
| NAE1 | sinah | SIAH1 | PUB13 | TCEB2 |
| UBA6 | SIAH2 | MOT2 | PUB44 | ASB1 |
| UBA5 | Mdm2 | BOI | SPL11 | SPSB4 |
| SAE2 | Trim27 | TFB3 | PPIL2 | ASB9 |
| uba-1 | Ring1 | TRIM13 | UBE4A | ASB7 |
| AXR1 | Trim15 | TRIM4 | OsI_04551 | MED8 |
| rfl-1 | Trim8 | March4 | PUB18 | TMF1 |
| UBE2K | Diap2 | AIRP1 | PRP19A | SOCS6 |
| ATG3 | nopo | RMA3 | OsI_22374 | ASB11 |
| ATG10 | Topors | RNF7 | PUB54 | SOCS4 |
| UFC1 | Trc8 | RNF220 | PUB56 | SOCS7 |
| RAD6 | Sce | TRIM6-TRIM34 | OsI_20111 | ASB12 |
| UBC6 | Bmi1 | PIB1 | OsI_15850 | NEURL2 |
| UBC8 | neur | March6 | PUB29 | SOCS3 |
| UBE2E1 | CG9014 | SINAT4 | PUB25 | SPSB2 |
| ben | Rchy1 | RMD5 | Os12g0161100 | ASB15 |
| UBC9 | Trim24 | RMA2 | CHIP | RAB40C |
| eff | tna | SAN1 | Os04g0418500 | SOCS2 |
| lwr | Trim21 | RNF6 | OsJ_07085 | Lrrc41 |
| UBC13 | elgi | FLY1 | OsJ_15959 | ASB4 |
| UBC5 | RNF152 | ATL42 | PUB9 | VHL |
| CDC34 | hiw | STE5 | LOC_Os10g03440 | lrr-1 |
| RCE1 | Roc1b | ATL14 | OsI_08535 | CISH |
| UBC22 | Ltn1 | SUD1 | CERBERUS | ASB6 |
| UBC4 | Trim37 | SLX5 | STUB1 | gus |
| UBE2G1 | Roc1a | TRIM36 | ARC1 | zif-1 |
| ubc-3 | Siah1a | AIRP2 | OsI_07564 | SPSB1 |
| Ube2a | Trim41 | NHLRC1 | PUB63 | SOCS5 |
| UBE2U | sel-11 | LUL1 | PUB23 | WSB2 |
| UBE2W | nhl-1 | RAD16 | CYP65 | LRR1 |
| UBE2E3 | F54B11.5 | RNF133 | bg55 | ASB8 |
| Bruce | Rnf138 | ATL73 | PUB38 | SOCS1 |
| Ube2l6 | F43G6.8 | XBAT33 | NOPPERABO1 | GPR75-ASB3 |
| UBC18 | F42G2.5 | SH3RF1 | Chi | FEM1B |
| ubc11 | apc-11 | HLTF | PUB41 | WSB1 |
| Ube2g2 | Neurl1b | ARI16 | PRPF19 | CORO7 |
| UBE2D4 | Amfr | KEG | PRP19B | ASB2 |
| UBC10 | F16A11.1 | TRIM2 | CDC26 | MMS22 |
| Ubc2 | F08G12.5 | MSL2 | ANAPC7 | ATG5 |
| UBC30 | prx-12 | RGLG2 | APC1 | HOXB4 |
| UBC3 | Trim56 | TRIM74 | ANAPC13 | fem-1 |
| UBE2R2 | Trim16 | MIEL1 | SWM1 | DCAF17 |
| UBE2S | Trim10 | NSMCE2 | CDC16 | RICTOR |
| UBE2B | Trim12a | PPARG | APC2 | DCAF15 |
| UBE2Z | Trim30a | mib-1 | DOC1 | DCAF16 |
| UBE2Q2 | BFAR | ZNRF1 | ANAPC11 | AHR |
| UBE2C | Trim31 | TRIM46 | APC5 | CRT10 |
| UBE2F | Rag1 | ZMIZ1 | APC4 | TRPC4AP |
| UBE2I | March8 | LUL3 | APC9 | ARNT |
| UBE2H | Pml | Y38F1A.2 | ANAPC1 | C14B1.3 |
| UBE2J2 | March2 | RBX1A | CDC23 | mec-15 |
| UBE2L3 | PIAS2 | AIP2 | ANAPC5 | At1g80440 |
| UBE2T | RNF213 | TRIM50 | MND2 | FBXO10 |
| hus5 | Trim11 | TRIM62 | CDC27 | SON1 |
| Ube2q1 | RNF167 | At5g22750 | ANAPC10 | Fbxo32 |
| rhp6 | Peli1 | RLIM | ANAPC4 | fbxl-1 |
| UBE2J1 | Ccnb1ip1 | RNF181 | CDC20 | FBXL2 |
| ubc-1 | TRAF3 | March11 | fzr-1 | AFR |
| UBC7 | Rnf41 | gei-17 | Fzr1 | EBF1 |
| COP10 | Mib2 | RNF4 | CDH1 | FBXL7 |
| UBE2O | DTX3 | TRAIP | mfr1 | DOR |
| UBE2N | Rnf148 | ZSWIM2 | fzy | FBXO8 |
| ubc-18 | RNF24 | RNF25 | bath-42 | COI1 |
| UBC12 | ZNRF3 | DTX3L | BT1 | pof12 |
| RCE2 | MYCBP2 | ATL31 | At5g48510 | MAX2 |
| UBC29 | Trim63 | MNJ8.6 | NPR3 | At2g44130 |
| UBC35 | Trim25 | ZMIZ2 | BPM5 | pof9 |
| UBC28 | March1 | RNF135 | BT5 | pof3 |
| UBC34 | Trim71 | RNF123 | NPR4 | FBX5 |
| UBE2M | Mid1 | At5g37890 | BT2 | pof5 |
| UBC36 | Rnf128 | RNF185 | BPM4 | SKP2 |
| UBC1 | RNF170 | RNF10 | BPM3 | CDCA3 |
| BIRC6 | TRIM3 | TRIM68 | bath-40 | FBXL17 |
| SCE1 | Trim39 | T02C1.1 | TRAF1 | pof13 |
| UBC5B | Lnx1 | At5g38070 | kel-8 | Fbxl20 |
| UBE2E2 | RNF217 | DTX2 | KLHL18 | TULP9 |
| let-70 | RNF130 | ORTH1 | At1g21780 | At5g42350 |
| PEX4 | Syvn1 | brl2 | klhl10 | ADO1 |
| ubc-25 | RNF150 | ATL23 | kel | ROY1 |
| MMS2 | Pja2 | brl1 | dbo | MDM30 |
| morgue | D7Ertd443e | ZNF645 | bath-41 | YLR224W |
| UBE2D1 | Rfpl4 | BRG1 | GAN | ETP2 |
| UBE2D2 | RNF182 | LRSAM1 | BTBD9 | ETP1 |
| UBE2D3 | Pex2 | ZNRF2 | KBTBD13 | HRT3 |
| UBE2V2 | March10 | At5g48655 | SPOP | DIA2 |
| UEV1A | RNF5 | TRIM72 | KLHL42 | AFB2 |
| UEV1B | Rnf212 | dsc1 | KBTBD7 | FBXO17 |
| uev-1 | Rnf34 | PELI3 | RCBTB1 | GRR1 |
| UEV1D | TRIM51 | IRF2BP1 | KLHL21 | RAD7 |
| UEV1C | PEX10 | At5g53910 | KLHL12 | Fbxw15 |
| UBE2V1 | Trim9 | SH3RF3 | BT3 | FBXO27 |
| STP22 | CBLL1 | XBAT32 | KLHL9 | RCY1 |
| TSG101 | TRIM26 | RNF139 | KLHL7 | DAS1 |
| SAP5 | TRIM40 | RNF113B | KLHL17 | skpt-1 |
| Rabex-5 | RFPL4A | RNFT1 | KLHL22 | pof2 |
| TNFAIP3 | Rnf40 | RNF187 | KBTB1 | FBL16 |
| KCTD7 | CHFR | RC3H2 | KLHL11 | Fbxo2 |
| DCUN1D3 | TRIM73 | CNOT4 | mel-26 | pof7 |
| At3g12760 | BRAP | RFFL | At1g01640 | FBL17 |
| DCUN1D5 | OSTM1 | slx8 | KLHL3 | At5g49980 |
| DCN1 | RBX1 | ATL9 | KLHL40 | CDC4 |
| DCUN1D1 | March7 | At2g34000 | KLHL41 | SKIP20 |
| DCUN1D2 | RNF43 | ARI9 | KLHL8 | ntc |
| HECW2 | PPARA | DRIP2 | KLHL13 | TIR1 |
| D2085.4 | BIRC7 | WAVH1 | KLHL2 | pall |
| NEDD4L | VPS18 | CESA6 | keap1a | EID1 |
| HERC4 | ZNRF4 | RKP | KLHL20 | FBXL21 |
| HERC3 | MGRN1 | CIP8 | KEAP1 | fbxa-196 |
| pub3 | TTC3 | OsI_05393 | KCTD13 | fbxa-167 |
| SMURF2 | DTX4 | rhp18 | KCTD11 | At5g48990 |
| TOM1 | BIRC2 | OsI_05734 | TNFAIP1 | CPR30 |
| ITCH | TRIM59 | RIE1 | btb2 | FBXO11 |
| UPL7 | TRIM23 | RHA2B | D2045.8 | FBXO42 |
| HECTD3 | RNF103 | BRG2 | C17F4.8 | ZC3HC1 |
| UPL2 | RNF11 | OsI_08453 | BPM1 | FBXL3 |
| HACE1 | RNF114 | At1g74370 | BCL6B | FBXO6 |
| Wwp1 | PIAS3 | RMR2 | BPM2 | FBXO25 |
| NEDD4 | RNF13 | OsI_17037 | ZC239.15 | Fsn |
| Smurf1 | RNF208 | At1g68180 | ZBTB18 | GID2 |
| HECTD4 | TRAF2 | At1g67180 | KCTD6 | FBX14 |
| G2E3 | RNF20 | At1g66650 | EOL2 | FBXO4 |
| UBE3D | RNF180 | ORTH5 | btb1 | FBXL5 |
| RSP5 | TMEM129 | ORTH4 | ARIA | YDR306C |
| HUL4 | PIAS1 | ARI8 | NACC1 | SNE |
| UFD4 | TRIM6 | OsI_18510 | btb3 | FBXO18 |
| PF3D7_0704600 | TRIM54 | OsI_27723 | inso-1 | MFB1 |
| UPL5 | TRIM5 | COP1 SUPPRESSOR 1 | bath-15 | YDR131C |
| HUL5 | TRIM22 | ORTH2 | KCTD5 | FBXL19 |
| HERC6 | TRIM47 | ORTH3 | RHOBTB1 | Ppa |
| HERC5 | NFX1 | HUB2 | RHOBTB2 | SAF1 |
| HERC1 | RNF26 | At1g50440 | btb-20 | FBXL14 |
| UPL4 | MKRN1 | ATL78 | rdx | FBXO44 |
| Ube3a | RNF122 | ATL76 | RPT3 | Kdm2 |
| WWP2 | TRIM45 | ATL47 | ETO1 | fsn-1 |
| UPL6 | DTX1 | rpm-1 | EOL1 | fbxa-215 |
| Smurf | March3 | ATL15 | ABTB1 | fbh1 |
| AREL1 | RNF38 | At1g18760 | BPM6 | AFB3 |
| eel-1 | ZK637.14 | C02B8.6 | B0281.5 | FBXL12 |
| pub1 | rbx-1 | F20D23.17 | KCTD21 | fbxa-210 |
| CG5604 | HOS1 | apc11 | RHOBTB3 | dre-1 |
| HECTD1 | BARD1 | rnf-1 | CUL7 | GRH1 |
| TRIP12 | ASR1 | RHA2A | CUL4B | FBXO40 |
| pub2 | ATL41 | C11H1.3 | cul-1 | pof6 |
| UBE3B | MNAT1 | At1g14260 | Cul4 | fbxa-115 |
| HECW1 | TRAF5 | At1g12760 | CUL4A | EBF2 |
| UPL3 | ULS1 | At1g10650 | CUL3A | T10E9.1 |
| wwp-1 | NFI1 | DRIP1 | RTT101 | mfb-1 |
| HECTD2 | At2g44330 | RING1B | CUL5 | FBXO9 |
| Su(dx) | RNF186 | BAH1 | CUL3 | At1g15670 |
| Ubr5 | At2g44410 | rfp1 | CUL3B | FBXW10 |
| HUWE1 | PJA1 | RSL1 | CDC53 | FBXO30 |
| hyd | TRIM32 | lin-41 | CUL2 | SKIP32 |
| UBE3C | HUB1 | rnf-121 | CUL1 | CCNF |
| HERC2 | HRT1 | GW2 | ANAPC2 | D3 |
| PRT6 | RFI2 | Os09g0434200 | DDB1B | pof14 |
| UBR4 | RNF8 | OsJ_19457 | DDB1A | FBXO45 |
| AURKA | RGLG1 | P0034C09.30 | ddb-1 | T07E3.4 |
| CADPS2 | ATL6 | PTB1 | DDB1 | FBXL15 |
| HDAC4 | PSH1 | rnf-5 | MMS1 | SLF3 |
| RASD2 | HRD1 | SBP1 | pic | SLF6 |
| Pdlim2 | MID2 | TR1 | dtl-a | FBXO7 |
| NSP1 | At3g05250 | CPN1 | EED | FBXO3 |
| RWDD3 | At3g05545 | pli1 | POC1B | UFO |
| EP300 | At3g06330 | ICP0 | RBBP5 | fsd |
| KAT2B | PIAS4 | RHE1 | TLE2 | pof8 |
| CREBBP | T1B9.13 | rnf12-a | GNB2 | VBF |
| FUS | DMA2 | RMA1H1 | DWA2 | FBXL6 |
| Ufl1 | RKR1 | rbrA | DTL | FBXO22 |
| SART1 | RNF113A | XELAEV_18034635mg | RBBP4 | FBXO31 |
| Cbx4 | At3g09760 | LAP | WDR59 | slf-S5 |
| E4F1 | LOG2 | ubr11 | DCAF4 | FBXO15 |
| MALT1 | BRG3 | ari-1 | RP11-574F21.3 | FBXO28 |
| rad60 | MMS21 | rad8 | WDR12 | slf-S2 |
| ZFP91 | ATL2 | brc-1 | cdt-2 | ADO3 |
| IRF2BPL | CST9 | madd-2 | TLE1 | FBXL4 |
| CRBN | PDZRN3 | C45G7.4 | WDR55 | FBXO33 |
| STC1 | BTS | rfp-1 | PHIP | ftr-1 |
| NAM7 | IRC20 | TRIM35 | DCAF10 | sel-10 |
| KCMF1 | ATL62 | UBR2 | DCAF5 | Btrc |
| UCHL1 | RAD5 | TRIM28 | DDB2 | pof11 |
| RANBP2 | SSL1 | PHRF1 | DCAF11 | pof1 |
| RNF19B | RNF146 | Ubox5 | NUP43 | FBXW11 |
| ARIH2 | XBAT35 | COP1 | KATNB1 | ebi |
| ARIH1 | RC3H1 | RFWD2 | GRWD1 | lin-23 |
| RNF19A | SHPRH | TRAF7 | RBBP7 | FBXW2 |
| RNF216 | PRT1 | RIN2 | TLE3 | slmb |
| RNF14 | At3g29270 | RIN3 | NLE1 | pop1 |
| RNF144B | TUL1 | CBLB | THO6 | fbxA |
| RNF144A | MDM4 | BIRC3 | MSI4 | ago |
| CUL9 | RDUF1 | CBL | AMBRA1 | FBXW5 |
| RBCK1 | MUL1 | XIAP | BRWD1 | Fbxw7 |
| RNF31 | F13I12.210 | RNF125 | WDR76 | TBL1XR1 |
| PARK2 | SIS3 | RNF111 | SMU1 | fbxw1 |
| KMT2A | T17F15.60 | RNF115 | WDR53 | TBL1X |
| KMT2B | TRIM17 | RAD18 | CIAO1 | pof10 |
| KDM2B | SSM4 | RNF126 | cdt2 | MET30 |
| AIRE | RNF121 | BRIZ1 | DWA3 | ELA1 |
| msc1 | LUL2 | BRIZ2 | PWP1 | UFO1 |
| MAP3K1 | DZIP3 | UHRF1 | WDTC1 | FBXW4 |
| ING4 | NSMCE1 | UHRF2 | WDR26 | FBXW8 |
| Os11g0629300 | SDIR1 | RBBP6 | DCAF7 | ASK9 |
| Os10g0445400 | AtRZF1 | PCGF1 | RACK1 | ASK3 |
| P0666G10.111 | RFWD3 | COMMD3-BMI1 | WDR5B | ASK5 |
| Os07g0673200 | DMA1 | RING1A | WDR82 | SSK1 |
| BBI1 | BB | Os02g0540700 | DCAF1 | SkpA |
| SIZ1 | Pit1 | PUB21 | DCAF13 | SKP1A |
| XB3 | RMA1 | NOSIP | l(2)dtl | ASK4 |
| EL5.3 | RNF141 | ufd-2 | SNRNP40 | skp1 |
| Os02g0301000 | UBR1 | PUB4 | BRWD3 | SKP1B |
| OsHRZ1 | VPS41 | Os04g0686000 | PRL1 | RNF168 |
| UPL1 |  |  |  |  |
