# Supplementary material for: Ubiquitination and ALL: Identifying FBXO8 as a prognostic biomarker and therapeutic target
Source: Front Immunol. 2025 May 1;16:1554231. doi: 10.3389/fimmu.2025.1554231 (PMC12078231; doi:10.3389/fimmu.2025.1554231)
Supplement: Supplementary Table 2 — The coefficient of final 9-gene signature formula. [file Table2.docx]

Supplemental Table 2. The coefficient of final 9-gene signature formula

| Gene | coefficient |
| --- | --- |
| ATL2 | 1.41171516095683 |
| MKRN1 | -0.678750778393132 |
| FBXW8 | -0.393423650100273 |
| FBXO8 | -0.588471615823997 |
| DCAF16 | -0.461894935538824 |
| WSB1 | -0.821762477759895 |
| CHFR | -0.581877953893865 |
| MDM2 | 0.100401623761693 |
| SOCS2 | -0.0110451352836066 |
