# Supplementary material for: Ubiquitination and ALL: Identifying FBXO8 as a prognostic biomarker and therapeutic target
Source: Front Immunol. 2025 May 1;16:1554231. doi: 10.3389/fimmu.2025.1554231 (PMC12078231; doi:10.3389/fimmu.2025.1554231)
Supplement: Supplementary Table 3 — Primer sequences used in qPCR analysis. [file Table3.docx]

Supplemental Table 3 Primer sequences used in qPCR analysis.

| Name | Sequence |
| --- | --- |
| hFBXO8-Forward | AGUAGAAUCCUGGAAAGGAGATT |
| hFBXO8-Reverse | TCCTTCCTGTTCTTTCGATTTCC |
| hGAPDH-Forward | GGAGCGAGATCCCTCCAAAAT |
| hGAPDH-Reverse | GGCTGTTGTCATACTTCTCATGG |
| mFbxo8-Forward | TGGCAAGACCTCGCTAATGAT |
| mFbxo8-Reverse | TGGACATAAAGTAGCTCACTCCC |
| mβ-actin-Forward | GTGACGTTGACATCCGTAAAGA |
| mβ-actin-Reverse | GCCGGACTCATCGTACTCC |
