# Supplementary figures and images for: Ubiquitination and ALL: Identifying FBXO8 as a prognostic biomarker and therapeutic target
Source: Front Immunol. 2025 May 1;16:1554231. doi: 10.3389/fimmu.2025.1554231 (PMC12078231; doi:10.3389/fimmu.2025.1554231)

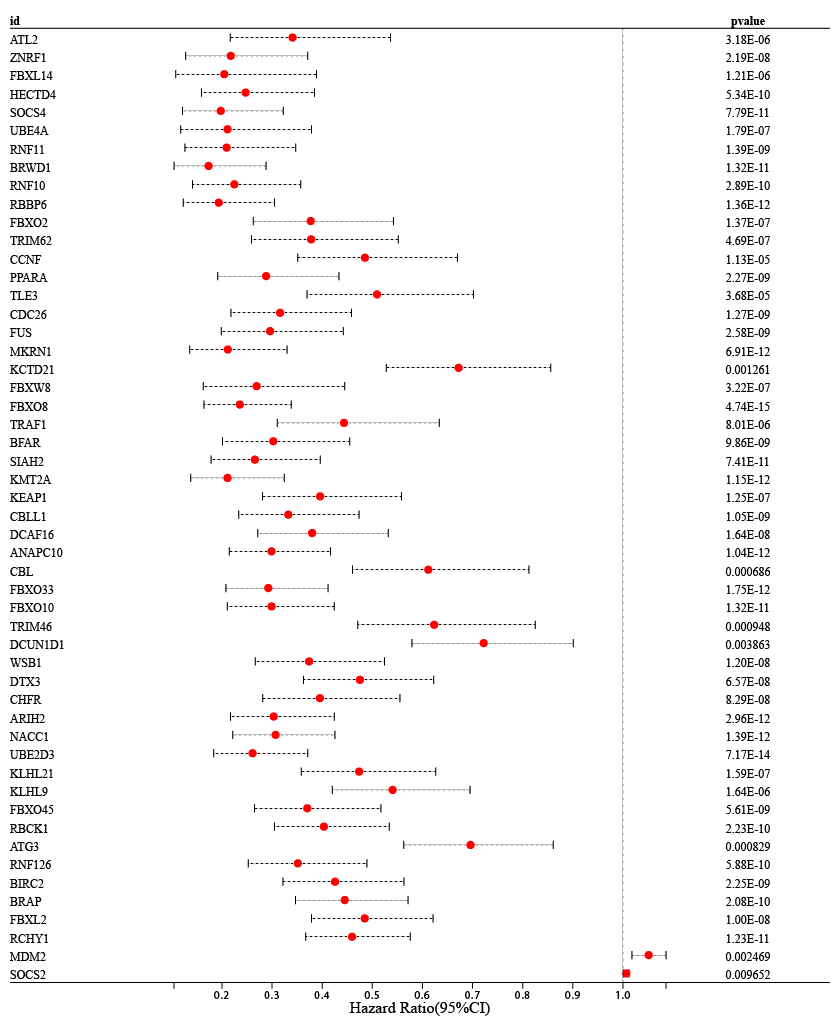

Supplement: Supplementary Figure 1 — Univariate Cox analysis results of genes significantly associated with the prognosis of ALL patients. [file Image1.tif]
